# Assessment of Oxaliplatin-Loaded Iodine Nanoparticles for Chemoradiotherapy of Human Colorectal Cancer (HT-29) Cells

**DOI:** 10.3390/polym14194131

**Published:** 2022-10-02

**Authors:** Naser Rasouli, Daryoush Shahbazi-Gahrouei, Simin Hematti, Behzad Baradaran, Roya Salehi, Jaleh Varshosaz, Abbas Jafarizad

**Affiliations:** 1 Department of Medical Physics, School of Medicine, Isfahan University of Medical Sciences, Isfahan 8174673461, Iran; 2 Department of Radiooncology, School of Medicine, Seyyed Al-Shohada Hospital, Isfahan University of Medical Sciences, Isfahan 8174673461, Iran; 3 Immunology Research Center, Tabriz University of Medical Sciences, Tabriz 5165665931, Iran; 4 Drug Applied Research Center, Department of Medical Nanotechnology, School of Advanced Medical Sciences, Tabriz University of Medical Sciences, Tabriz 5165665931, Iran; 5 Department of Pharmaceutics, School of Pharmacy and Pharmaceutical Sciences, Isfahan University of Medical Sciences, Isfahan 8174673461, Iran; 6 Department of Chemical Engineering, Sahand University of Technology, Tabriz 5165665931, Iran

**Keywords:** iodine nanoparticles, radiotherapy, chemotherapy, oxaliplatin, HT-29 colorectal cancer

## Abstract

Colorectal cancer is highly prevalent worldwide and has significant morbidity and mortality in humans. High-atomic-number nanoparticles such as iodine can act as X-rays absorbers to increase the local dose. The synthesis and fabrication of oxaliplatin-loaded iodine nanoparticles, their characterization, cell toxicity, radiosensitivity, cell apoptosis, and cell cycle assay in human colorectal cancer (HT-29) cells are investigated. Results show that the synthesis of a new iodine nanoparticle, polymerized triiodobenzene coated with chitosan and combined with oxaliplatin as a chemotherapeutic drug, performed well in vitro in an intracellular radiosensitizer as chemoradiotherapy agent in HT-29 cell lines. Findings also show that the INPs alone have no impact on cell cycle development and apoptosis. In contrast, oxaliplatin-loaded INPs along with 2 and 6 MV radiation doses produced more apoptosis. The interaction of INPs with mega-voltage photon energies is the cause of a major radiosensitization enhancement in comparison to radiation alone. Furthermore, results show that INPs may work as radiosensitization nanoprobe agents in the treatment of HT-29 cells due to their effect on increasing radiation dose absorption. Overall, iodine nanoparticles may be used in the treatment of colorectal cancers in clinical studies.

## 1. Introduction

Cancer has priority over heart disease as the leading cause of death in developed countries in 2020 [1]. Human colorectal cancer is the fourth most frequently diagnosed type of cancer worldwide, manifesting as a malignant neoplasm in the colon or rectum mucosa [2].

Radiotherapy has for a long time been a standard treatment strategy for different cancers, such as colorectal cancer. Although an adequate response to radiation therapy is achieved during the primary phase of cancer treatment, many cancer patients have radiation resistance [3]. Today, radiation therapy evaluations based on the enhancement efficacy of treatment using radiosensitizers are applicable.

High-atomic-number materials are an efficient approach to improve radiation therapy effectiveness. Thus, metal nanoparticles may increase the contrast between the tumor and surrounding tissues. This is a reason why they are called radiosensitizers and can improve the efficacy of radiation therapy [4,5]. It is known that if tumors are irradiated with X-rays while accompanied with absorber materials, the irradiated dose can increase locally and the effectiveness of radiation therapy is improved.

Gold nanoparticles demonstrated early promise in test animals, annihilating 50% of advanced gliomas [6]. Recently, scientific efforts have been made to apply high-atomic-number materials such as gold or silver nanoparticles as radio enhancement under in vitro and in vivo conditions [7]. However, clinical uses of the mentioned nanoparticles have slowed significantly over the last several years due to several significant disadvantages. For instance, some disadvantages of gold nanoparticles include required high doses of IV injection, being highly colored, long blood half-life, poor clearance, and high cost.

Therefore, iodine, which is almost colorless, non-toxic, a good absorber of X-rays due to photoelectric effects, less costly, and has reasonable clearance can compensate for major drawbacks of the gold nanoparticles and is a good substitute for gold [8,9,10,11].

In a study by Adam et al. in 2016, radiation with an energy of 80 keV generated by synchrotron after intravenous administration of standard iodine contrast media was applied [12]. In another study, iodine nanoparticle radiotherapy was used for treatment of human glioma in mice and resulted in a median life extension of more than double that of radiation therapy alone [13]. Currently, iodine is a suitable candidate for enhancing radiotherapy because of its outstanding properties including cancer cell internalization and biocompatibility. 

Oxaliplatin is a platinum compound of the third generation that is less toxic and more tolerable than other platinum drugs [14,15]. Oxaliplatin was chosen in the present study due to its useful clinical results for treating head and neck tumors [16], which were verified in vitro using a specific line of human colorectal cancer cells (HT-29).

To the best of the authors’ knowledge, there is no published work on the application of oxaliplatin-loaded iodine nanoparticles as radiosensitization in chemoradiotherapy of HT-29 cells. Herein, for the first time, the anti-cancer effect of oxaliplatin-loaded iodine nanoparticles in chemoradiotherapy and its possible mechanisms are investigated.

## 2. Materials and Methods

### 2.1. Chemicals

All chemical compounds, including amino PEG (Polyethylene glycol), oxaliplatin, iohexol, sodium periodate, carbohydrazide, sodium borohydride, chitosan, HT-29 cell line, DMSO, and MTT, were prepared from Sigma-Aldrich, Munich, Germany.

HT-29 cells were obtained from Pasteur Institute (Tehran, Iran). The cells were cultured in RPMI 1640 medium consisting of 10% fetal bovine serum (FBS) and 1% streptomycin (Sigma-Aldrich, Germany). Then, they were stored in a humidified incubator at 37 °C with 5% CO_2_.

### 2.2. Synthesis of Iodine Nanoparticles

The synthesis of iodine nanoparticles coated with amino PEG (INP1) and iodine nanoparticles coated with chitosan (INP2) was carried out according to the method reported by Hainfeld et al., with some modifications [13]. In brief, in a typical preparation, 108.8 mg of iohexol was oxidized with 34 mg of sodium periodate for 30 min under stirring magnetic conditions and maintained to dry. The yielding material was dissolved in water and polymerized with 5.36 mg of carbohydrazide. Then, 400 mg of 3 kDa amino PEG was added to the cross-linked particles and remained for 12 h. Sodium borohydride (7.5 mg) was added and allowed to react for 3 h. The solutions were dialyzed and freeze-dried. Furthermore, iodine nanoparticles coated with chitosan were synthesized by using 400 mg of chitosan, 435.2 mg of iohexol, 136 mg of sodium periodate, 21.44 mg of carbohydrazide, and 30 mg of sodium borohydride.

### 2.3. FT-IR Analysis

The Fourier transformed infrared spectrophotometer, FTIR (Perkin-Elmer Life and Analytical Sciences, Inc., Waltham, MA, USA), was applied to obtain the FTIR spectrum and confirmed the structure of the fabricated nano-agent in the range of 4000 to 400 cm^–1^ to evaluate the chemical interaction between oxaliplatin and nanoparticles. For this purpose, nanoparticle suspensions with oxaliplatin were prepared as stated in Section 2.2. To obtain dried nanoparticles, their suspensions with/without entrapment of oxaliplatin were lyophilized by a freeze-drying system.

### 2.4. Characterization of Iodine Nanoparticles

Zeta potential (Zetasizer^®^ 3000HS (Malvern Instruments, London, UK) and size of INPs were measured by dynamic light scattering (DLS) and laser doppler anemometry (LDA). For size measurements, samples were diluted in water and measured for a minimum of 3 min. Raw data were subsequently correlated to mean hydrodynamic size using an accumulation assay. For zeta potential measurements, deionized water was added to the samples and they were measured in automatic mode. All measurements were taken three times.

The morphology of particles was obtained by transmission electron microscopy (CM12 Philips, Eindhoven, Netherlands). Samples were stained with 2% phosphotungstic acid for 10 min, immobilized on copper grids, and dried overnight.

### 2.5. Entrapment Efficiency Spectrophotometric Assay

An appropriate amount of INP1 and INP2 nanocarriers was dissolved in separated vials, and a predetermined amount of oxaliplatin solution was added to each vial and kept in dark conditions under stirring overnight (nanoparticle to oxaliplatin feed ratio was 5:1). Then, oxaliplatin-loaded nanoparticles (1 mL) were centrifuged, and the supernatant was removed. The oxaliplatin amount in the supernatant was obtained by UV/Vis spectrophotometer at 510 nm [17]. The standard curve of oxaliplatin was performed in concentrations in the range of 5 to 60 µg/mL. The entrapment efficiency was obtained by the following formula:

Entrapment efficiency (%) = [(Total Drug − Free Drug)/Total Drug] × 100(1)

### 2.6. In Vitro MTT Assays and Cytotoxicity (IC50)

The cell viability of the studied groups was analyzed by MTT (3-(4,5-dimethylthiazol-2-yl)-2,5 diphenyl tetrazolium bromide) assay [18]. A number of 5 × 10^3^ cells/well were seeded in a 96-well cell culture plate. The cells were incubated in a culture medium for 24 h at 37 °C (5% CO_2_). After one day, the cells were treated with different concentrations for 48 h. Then, cells were incubated using 50 μL/well of phosphate-buffered saline (PBS) containing 1 mg/mL MTT for an additional 4 h. The intracellular formazan crystals that reduced the tetrazolium salts present only in metabolically active cells were solubilized with dimethyl sulfoxide (DMSO). The absorbance of active cells was measured at 540 nm to assess the cell viability according to the following equation:

(2) $$\mathrm{Cell}\;\mathrm{viability}\%\;=\frac{\;\mathrm{Mean}\;\mathrm{absorbance}\;\mathrm{of}\;\mathrm{sample}\;-\;\mathrm{mean}\;\mathrm{absorbance}\;\mathrm{of}\;\mathrm{blank}}{\mathrm{Mean}\;\mathrm{absorbance}\;\mathrm{of}\;\mathrm{negative}\;\mathrm{control}\;-\;\mathrm{mean}\;\mathrm{absorbance}\;\mathrm{of}\;\mathrm{blank}}\times 100$$

Cell viability assay was performed for similar concentrations of treated cells. Irradiation of treated cells was performed using a linear accelerator (Primus, Siemens Ltd., Munich, Germany) with the protocol of source-to-surface distance (SSD) = 100 cm, field size = 25 × 25 cm^2^, and delivered total doses = 2 and 6 Gy with a dose rate = 200 MU min^−1^.

The cytotoxic effect of the cells (IC50) was measured microscopically with phase-contrast microscopy (100× magnifications) after treatment of cells at 24 h.

### 2.7. Cell Apoptosis Study

The apoptotic effects of different formulations on HT-29 cell lines were assessed using the Annexin V-FITC Apoptosis Detection Kit (Abcam plc, Cambridge, UK) [19]. Cells at a density of 1 × 10^5^ cells/well in 1 mL of medium were seeded in 6-well plates and treated after 24 h. The collected cells were stained with Annexin V-FITC and propidium iodide (PI; provided in the kit) in the dark and analyzed by flow cytometry apparatus (BD, Franklin Lakes, NJ, USA).

### 2.8. Cell Cycle Determination

Determination of cell apoptotic peaks was performed using flow cytometry [20]. The cells were seeded on 100 mm dishes, grown in a cell culture medium, and supplemented with 10% fetal bovine serum (FBS). After treatment with different formulations for 24 h, they were collected, trypsinized, washed with PBS, and fixed by adding 2 mL of cold 70% ethanol, followed by storage at 4 °C. After fixation, they were washed, centrifuged, and resuspended in 0.05 mg/mL propidium iodide, 100 U/mL RNase. Then, the cells were incubated for 30 min at room temperature in the dark and analyzed using a flow cytometer (FLOWJo software, Tree Star, Inc., Ashland, OR, USA). Cell cycle results were obtained with CellQuest software. They were analyzed again using ModFit software (Verity Software House, Topsham, Maine, USA). At the same time, negative controls were fabricated.

### 2.9. Statistical Analysis

All experiments were performed three times, and the statistical significance analysis was performed with IBM SPSS version 20 (IBM Crop. 2021. IBM SPSS Statistics for Windows, version 26.0. NY, EUA). One-way Analysis of Variance (ANOVA) test is considered to verify the statistically significant cytotoxicity. Statistical significance was defined at a *p <* 0.05.

## 3. Results

### 3.1. Synthesis of Iodine Nanoparticles

**Figure 1:**
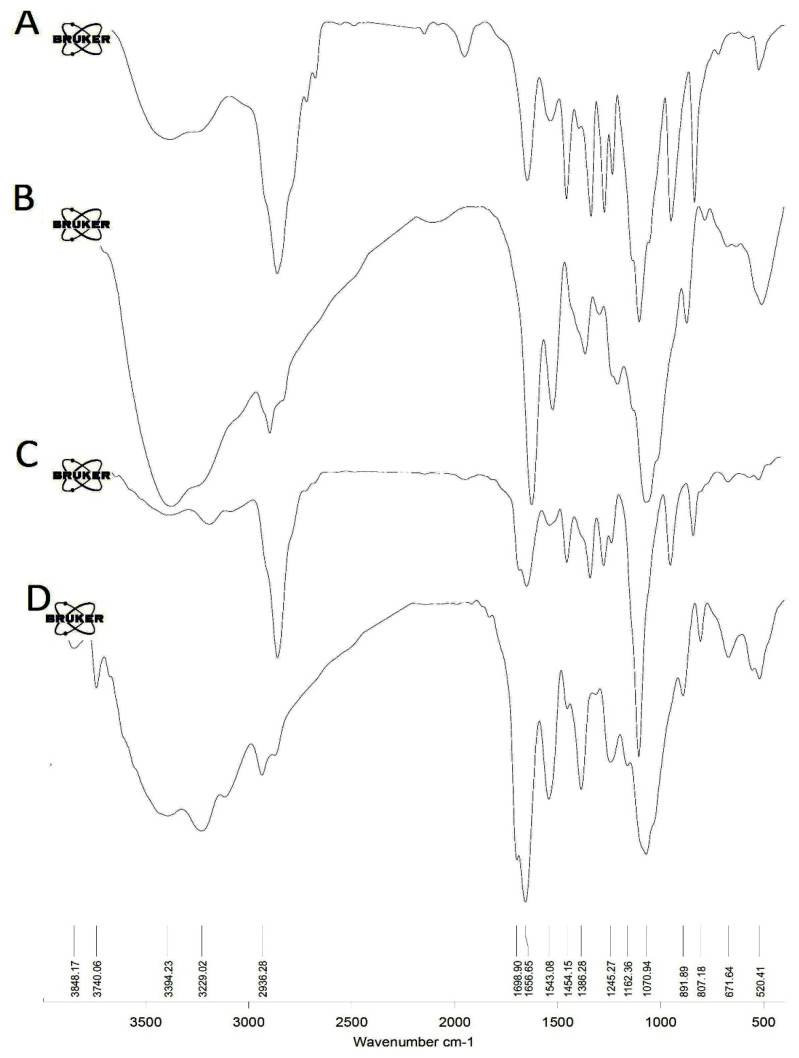
FTIR spectra for the synthesized iodine nanoparticles. (**A**) Amino-PEG iodine nanoparticles, (**B**) chitosan iodine nanoparticles, (**C**) oxaliplatin-loaded Amino-PEG/iodine nanoparticles, and (**D**) oxaliplatin-loaded chitosan/iodine nanoparticles.

FTIR spectra of iodine nanoparticles are shown in Figure 1A,B. The peaks at 3218 cm^−1^, 2884 cm^−1^, 1661 cm^−1^, and 1110 cm^−1^ were assigned to the stretching vibrations of the N-H, C-H, C=O, and C-O, respectively. Furthermore, peaks in the 1464 cm^−1^ and 1349 cm^−1^ regions represent the C-H deformation vibrations. In addition, peaks in the 1283 cm^−1^ and 1244 cm^−1^ regions correspond to the O-H bending vibrations (Figure 1A).

The peak at 3403 cm^−1^ is attributed to –OH group stretching vibrations. The appearance of a peak at 2924 cm^−1^ indicates the presence of aliphatic asymmetrical C-H stretching in the methylene group. The peaks at 1647 cm^−1^ and 1545 cm^−1^ are attributed to the C=O stretch and NH out of the plane of amides groups, respectively. The absorption peak of 1386 cm^−1^ was accompanied by the bending vibrations of OH. The peak at 1090 cm^−1^ was assigned for anti-symmetric stretching of the (C–O–C) bridge in Chitosan (Figure 1B).

In FTIR spectra of both oxaliplatin-loaded Amino-PEG/iodine nanoparticles (Figure 1C) and oxaliplatin-loaded chitosan/iodine nanoparticles (Figure 1D), the appearance of a new peak at around 1700 cm^−1^ was related to the O-C=O section in the oxaliplatin structure.

### 3.2. Morphology and Size of Iodine Nanoparticles

**Figure 2:**
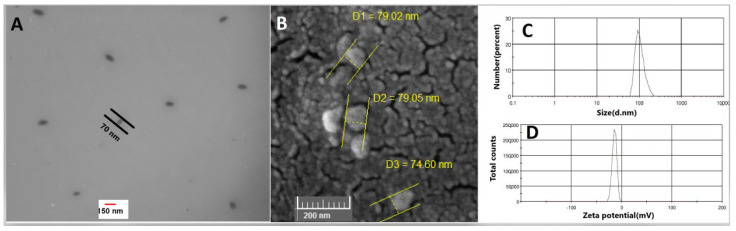
TEM (**A**) and SEM (**B**) photographs of iodine nanoparticles coated with amino-PEG (INP1). (**C**) Size distribution of iodine nanoparticles, (**D**) Zeta potential distribution of iodine nanoparticles.

**Figure 3:**
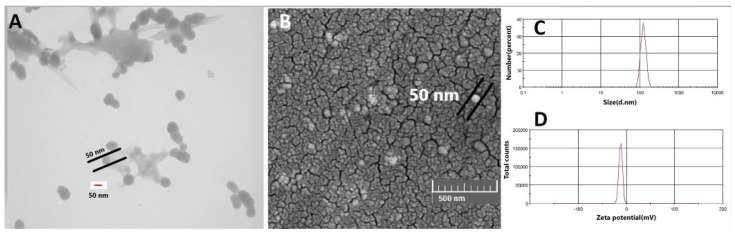
TEM (**A**) and SEM (**B**) photographs of iodine nanoparticles coated with chitosan (INP2). (**C**) Size distribution of iodine nanoparticles, (**D**) Zeta potential distribution of iodine nanoparticles.

The mean particle sizes of amino-PEG/iodine nanoparticles and chitosan/iodine nanoparticles INPs were 104 nm with PDI of 0.587 (Figure 2C) and 123 nm with PDI of 1 (Figure 3C), respectively. As Figure 2D and Figure 3D indicate, the zeta potential of nanoparticles showed a negative charge of about −14 mV and −12 mV, respectively. A solid structure and a circular shape of nanoparticles were obtained using TEM and SEM (Figure 2A,B and Figure 3A,B). Indeed, the size of iodine nanoparticles determined with electron microscopy was 70 nm and 50 nm, as indicated in Figure 2A and Figure 3A, respectively.

**Figure 4:**
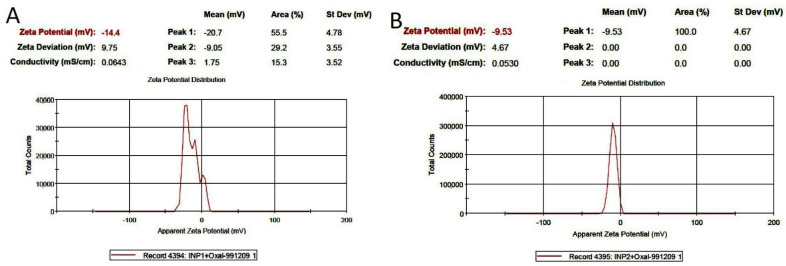
Zeta potential distribution of (**A**) Oxaliplatin-loaded Amino-PEG/iodine nanoparticles and (**B**) Oxaliplatin-loaded chitosan/iodine nanoparticles.

The entrapment efficiencies of oxaliplatin-loaded amino-PEG/iodine nanoparticles and oxaliplatin-loaded chitosan/iodine nanoparticles were determined to be 77% and 65%, respectively, using a UV/vis spectrophotometer at 591 nm. The surface charges of oxaliplatin-loaded amino-PEG/iodine nanoparticles and oxaliplatin-loaded chitosan/iodine nanoparticles were −14.4 and −9.53 mV, respectively (Figure 4).

### 3.3. Cytotoxicity Assay

**Figure 5:**
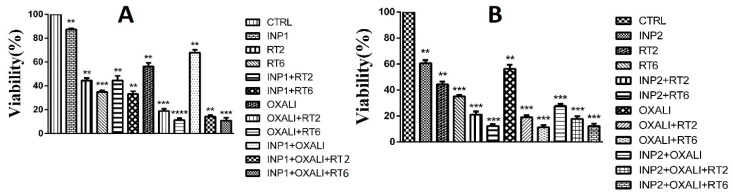
Inhibition of various formulations of HT-29 cell proliferation. Abbreviations: INP1, iodine nanoparticle 1. INP2, iodine nanoparticle 2. RT2, Radiotherapy at 2 Gy. RT6, Radiotherapy at 6 Gy. OXALI, Oxaliplatin. (**A**) Amino-PEG iodine nanoparticles; (**B**) chitosan iodine nanoparticles; ** *p*-value < 0.05, *** *p*-value< 0.01, and **** no significant.

In this study, the exponential HT-29 cells were treated with various formulations (Figure 5A,B), and the cell survival was measured by the MTT assay. INP1 and INP2 were used here at 1 mg/mL concentration and showed 85% and 60% cell viability across HT-29 cells, respectively. Oxaliplatin-loaded INP1 did not significantly reduce the cell viability of HT-29 cells compared to RT2, RT6, and oxaliplatin. Oxaliplatin-loaded INP2 significantly reduces the cell viability in HT-29 cells compared to oxaliplatin.

There was no significant difference in cell viability between RT2 and INP1 + RT2 (* *p* < 0.05). However, there was a significant difference in cell survival between INP1 + OX + RT2, INP1 + OX + RT6, INP2 + OX + RT2, INP2 + OX + RT6 with RT2, RT6, and oxaliplatin (* *p* < 0.05). Significantly all formulations were different in cell viability compared to the control group.

**Figure 6:**
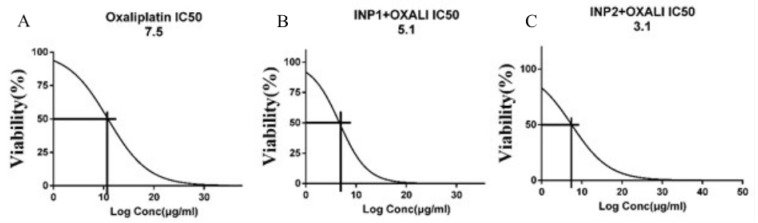
Inhibition effect of Oxaliplatin and INP1 + OXALI IC50 on HT-29 cells growth. IC50 was calculated for 3 formulations by MTT assay: (**A**) Oxaliplatin, (**B**) INP1 + OXALI, and (**C**) INP2 + OXALI. Abbreviations: INP1, iodine nanoparticle 1. INP2, iodine nanoparticle 2. OXALI, Oxaliplatin.

Fifty percent inhibition of different formulations (IC_50_) of oxaliplatin and oxaliplatin-loaded iodine nanoparticles was obtained. As shown in Figure 6A–C, the IC_50_ values for oxaliplatin and oxaliplatin-loaded iodine nanoparticles were 7.5 ± 1.1 μg/mL, 5.1 ± 0.6 μg/mL, and 3.1± 0.4 μg/mL, respectively. Oxaliplatin-loaded iodine nanoparticles 1 and 2 repressed the proliferation rate of HT-29 cells more effectively than oxaliplatin alone (*p* < 0.05).

### 3.4. In Vitro Apoptosis Assay on HT-29 Cells

**Figure 7:**
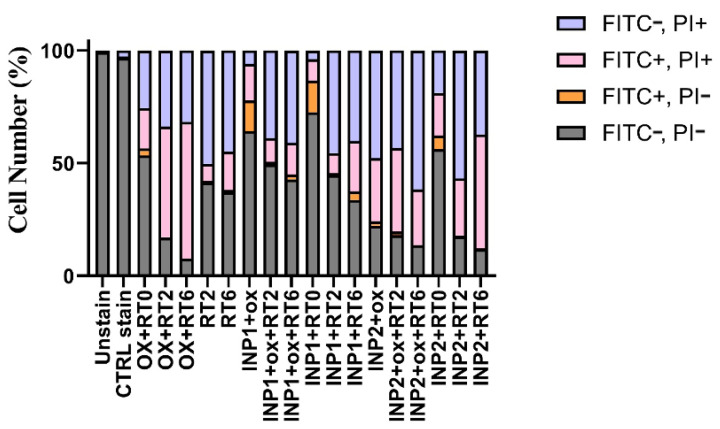
Quantitative results of apoptotic effects evaluated by Annexin V/FITC assay. Abbreviations: INP1, iodine nanoparticle 1. INP2, iodine nanoparticle 2. 0, Radiotherapy at 0 Gy. 2, Radiotherapy at 2 Gy. 6, Radiotherapy at 6 Gy. OX, Oxaliplatin.

Figure 7 illustrates the incident with no considerable change in the apoptotic rate of HT-29 cells (*p* > 0.05). INP1 + 6 displayed 3.81 ± 0.2% cell numbers in the first phase and 22.4 ± 1.9% in the late apoptotic phase. This figure shows that INP2 + 6 demonstrated 0.4 ± 0.024% and 50.6 ± 4% cell numbers in the early phase and end of the apoptotic phase, respectively.

Results of apoptosis assay show INP1 + OX + R2 enhanced cell proportion in apoptotic signal up to 50 ± 4.15% (*p* < 0.05). As can be seen from this figure, the percentage of the necrotic cells increased up to 57 ± 4.2% and 86 ± 7.6%, respectively, when covering HT-29 cells with INP1 + OX + RT6.

### 3.5. Cell Cycle Effects

**Figure 8:**
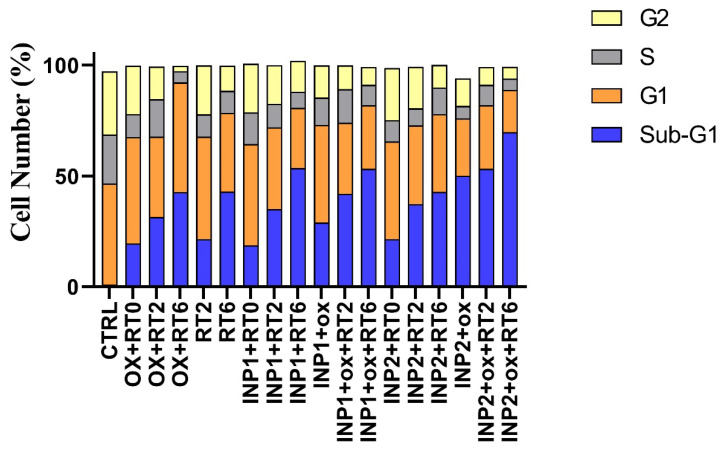
The effect of different formulations on HT-29 cell cycle profiles compared to control. Abbreviations: INP1, iodine nanoparticle 1. INP2, iodine nanoparticle 2. RT0, Radiotherapy at 0 Gy; RT2, Radiotherapy at 2 Gy; RT6, Radiotherapy at 6 Gy; and OX, Oxaliplatin.

The cell cycle analysis investigates the various stages of cell cycle and DNA duplication, including: G1, S, G2, and M. Cell cycle analysis was obtained using flow cytometry, and the results are presented in Figure 8. As shown in this figure, untreated HT-29 cells in the G0/G1, S, or G2/M phases showed normal growth conditions. The blank INP1 and INP2 showed small changes in cell cycle pattern in comparison with the control group (around 18.7–20% of cells arrested in sub-G1), which shows little toxicity to HT-29 cells. Free oxaliplatin showed around 19.6% Sub-G0/G1 arrest compared to the control group. However, in nano-formulation forms, INIP1 + OX and INIP2 + OO, around 29 and 50% Sub G0/G1 arrest were observed, which confirms the superior cytotoxicity of oxaliplatin in nano-formulations.

## 4. Discussion

Cancer remains a significant global health burden, and innovative chemoradiotherapy techniques are still required [21,22,23]. Radiation therapy is utilized in around 50% of all cancer therapies [24]; therefore, enhancing it would assist a large number of people. However, the physical radioenhancers previously mentioned have several disadvantages, including small-molecule contrast agents (such as iodine as a contrast medium and gadolinium chelates as contrast agent), rapid excretion, inability to penetrate tumors, rapid tumor washout, and high background in normal surrounding tissues at early time points following administration. High tumor-loading nanoparticles with lengthy blood half-lives accumulate in the liver, spleen, and other tissues consumed by macrophages. Many metal nanoparticles are strongly pigmented, requiring large amounts of IV infusion to achieve significant tumor concentrations, resulting in lasting skin pigmentation, weak whole-body clearance, and high expense. Iodine nanoparticles are non-toxic at high levels (7 g I/kg), colorless, highly X-ray absorbing, and organic (not non-degradable metal). They also have a long blood half-life (40 h), high uptake in tumors, and slow but consistent liver clearance (50% by six months, 70 percent by 12 months) [13].

In this study, the effect of two types of iodine nanoparticles (INP1 and INP2) in combination with oxaliplatin on human colon cancer cells (HT-29) was investigated. The size of nanoparticles determined with electron microscopy is between 50 and 70 nm and is comparable to previous studies. On the other hand, as in previous studies in mice, it had no detectable toxicity [25]. The particle size obtained from DLS measurement is rather larger than the results achieved from SEM and TEM in Figure 3, which may be related to the formation of pseudo-clusters and a surface hydration layer on samples under particle size measurement. Similar differences between the result of SEM and/or images and particle size measurement were also reported by other researchers [26,27].

Figure 5A,B demonstrate that at the lower size of INPs (50 nm of INP2 vs. 70 nm of INP1), there is a major difference in the cell-killing effect between INP2 + radiation and INP1 + radiation. Ghahremani and coworkers found that the size of the nanoparticles has a major role in radiosensitization: namely, ultrasmall size causes a high surface-to-volume ratio and can lead to an increase in the interaction of radiation beams and nanoparticles [28]. A major finding is that the sensitization rates depend upon the existence of oxaliplatin at low irradiated values. On the contrary, with a dose of 6 Gy, more than 85% of cells are killed in the presence of the oxaliplatin drug.

As the radiosensitizers have a predominate effect on radiotherapy efficacy, there is no major difference in cell survival between those treated with INP1-oxaliplatin and those treated with INP2-oxaliplatin. Both of them birradiated with different doses of radiation (Figure 5A,B). These results suggest that INPs–oxaliplatin–radiation leads to high-quality drug delivery and local consumption dose at MV photon energy. The study of Fathy et al. on chitosan-capped gold nanoparticles confirms the results of this study [29]. Figure 5A,B show that in terms of radiation sensitivity, there is a significant difference between INPs + oxaliplatin + RT6 and INPs + oxaliplatin + RT2, which shows the effect of radiation dose on cell death and increased treatment efficiency in terms of viability. However, the results obtained from INP2 + RT6 are more favorable than INP1 + RT6 in terms of cell death. In other words, INP2 nanoparticles were more effective than INP1 nanoparticles in increasing the radiation sensitivity of cells. The results of this study on the presence of INP2 combined with radiotherapy are comparable to the findings of Arab-Bafrani et al. regarding the effect of chitosan nanocomposite on increasing radiation sensitivity [30].

According to the results obtained (Figure 7 and Figure 8) from radiotherapy of cells with a dose of 2 Gy and in the absence of iodine nanoparticles, necrosis is observed, and the number of cells located in the FITC^−^, PI^+^ region (50%) is near to the viability results of MTT (around 50% mortality). On the other hand, the percentage of apoptotic cells was about 8%. The results of the MTT assay in this study show that about 60% of the cells were killed after radiotherapy, but necrosis was also observed among them. It is possible that a part of the cells is apoptotic or that both apoptosis and necrosis have been induced simultaneously. In 2 Gy radiotherapy, in the presence of INP2 (Figure 7), the percentage of apoptotic cells will increase, and the presence of nanoparticles has increased apoptosis, but necrosis is still predominant. In the combination of INP2 + OX + RT6, with the addition of oxaliplatin and increasing the radiation dose (Figure 7), necrosis will become more prevalent, from which it can be concluded that with increasing radiation dose and the presence of the drug, necrosis will prevail over apoptosis. The results of a study by Rostami et al. also confirm the results here, which show that in radiotherapy, necrosis will be the dominant mechanism for cell death [31].

Flow cytometry findings show the effects of different formulations on cell cycle progression in HT-29 cells (Figure 8). The Sub-G0/G1 area is of special interest in many toxicity studies that show the characteristics of apoptosis [32,33,34]. HT-29 cells that received radiation therapy only showed Sub-G0/G1 (21.5% in 2 Gy and 42.9% in 6 Gy), which is more dominant in 6 Gy radiation-treated groups. In cell treatment groups that received both chemotherapy and radiotherapy (INP1 + OX + RT2, INP1 + OX + RT6, INP2 + OX + RT2, and INP2 + OX + RT6), a synergistic effect was observed, which leads to a drastic increase in the percentage of the sub-G1 cell population. Totals of 42, 53, 53%, and 70% of cells were shifted to the Sub-G1 phase after treatment with INP1 + OX + RT2, INP1 + OX + RT6, INP2 + OX + RT2, and INP2 + OX + RT6, respectively.

When paired with the chemotherapeutic oxaliplatin, iodine nanoparticle irradiation significantly improved the efficacy of chemotherapy, leading to greater cell damage. Current optimization research should further improve the efficacy of iodine nanoparticle radiation before it can be successfully translated into the clinic.

## 5. Conclusions

In the presented work, novel oxaliplatin-loaded iodine nanoparticles are fabricated and applied to human colorectal cancer (HT-29) cells. The size and zeta potential of prepared nanoparticles were found to be 120 nm and −12 mV, respectively. This new nanoprobe represented the proliferation in HT-29 cells more effectively than oxaliplatin alone. Fabricated iodine nanoparticles also enhanced the efficacy of chemoradiotherapy when it was accompanied by the chemotherapy agent oxaliplatin. The high local dose of the tumor, which resulted in absorbing more X-ray photons during radiation therapy when using iodine, provided significant life extension compared to radiotherapy alone. Overall, it is a promising new nanoprobe for colorectal cancer (HT-29) cell treatment. 

## Data Availability

The data presented in this study are available on request from the corresponding authors.
